# Analysis of the cell surface layer ultrastructure of the oral pathogen *Tannerella forsythia*

**DOI:** 10.1007/s00203-012-0792-3

**Published:** 2012-01-25

**Authors:** Gerhard Sekot, Gerald Posch, Yoo Jin Oh, Sonja Zayni, Harald F. Mayer, Dietmar Pum, Paul Messner, Peter Hinterdorfer, Christina Schäffer

**Affiliations:** 1 Department of NanoBiotechnology, Vienna Institute of BioTechnology, Universität für Bodenkultur Wien, Muthgasse 11, 1190 Wien, Austria; 2 Christian Doppler Laboratory of Nanoscopic Methods in Biophysics, Institute for Biophysics, Johannes Kepler University, Altenbergerstrasse 69, 4070 Linz, Austria

**Keywords:** *Tannerella forsythia*, S-layer, Ultrastructure, Glycoprotein, Transmission electron microscopy, Atomic force microscopy

## Abstract

The Gram-negative oral pathogen *Tannerella forsythia* is decorated with a 2D crystalline surface (S-) layer, with two different S-layer glycoprotein species being present. Prompted by the predicted virulence potential of the S-layer, this study focused on the analysis of the arrangement of the individual S-layer glycoproteins by a combination of microscopic, genetic, and biochemical analyses. The two S-layer genes are transcribed into mRNA and expressed into protein in equal amounts. The S-layer was investigated on intact bacterial cells by transmission electron microscopy, by immune fluorescence microscopy, and by atomic force microscopy. The analyses of wild-type cells revealed a distinct square S-layer lattice with an overall lattice constant of 10.1 ± 0.7 nm. In contrast, a blurred lattice with a lattice constant of 9.0 nm was found on S-layer single-mutant cells. This together with in vitro self-assembly studies using purified (glyco)protein species indicated their increased structural flexibility after self-assembly and/or impaired self-assembly capability. In conjunction with TEM analyses of thin-sectioned cells, this study demonstrates the unusual case that two S-layer glycoproteins are co-assembled into a single S-layer. Additionally, flagella and pilus-like structures were observed on *T. forsythia* cells, which might impact the pathogenicity of this bacterium.

## Introduction

The periodontal pockets of humans harbor more than 500 bacterial species. Among them is a group of bacteria that constitute the “red complex”, comprising *Tannerella forsythia*, *Porphyromonas gingivalis*, and *Treponema denticola*, with the latter being strongly implicated in the onset of periodontitis (Socransky et al. 1998). Periodontitis is a chronic inflammation of the periodontium with multifactorial etiology. *T.* *forsythia* meets the criteria for being considered a periodontal pathogen (Socransky 1979) because of (1) its association with and increased levels in periodontitis (Socransky et al. 1998), (2) the evidence of host responses to its antigens (Bird et al. 2001; Yoo et al. 2007), (3) its ability to cause disease in animal models (Sharma et al. 2005; Kesavalu et al. 2007), and (4) the existence of distinct virulence factors that can contribute to the disease process (Sharma 2010).

*Tannerella forsythia* is an anaerobic Gram-negative bacterium belonging to the *Cytophaga*-*Bacteroidetes*-*Firmicutes* cluster of bacteria. It was initially named *Bacteroides forsythus* (Tanner et al. 1986) and later reclassified as *T.* *forsythia* (Sakamoto et al. 2002). Its cell surface is covered with a regularly arrayed surface (S-) layer (for review see Messner et al. 2010), and early electron microscopic investigations have shown the presence of an orthogonal S-layer lattice (Kerosuo 1988). SDS-PAGE of intact cells revealed that two high-molecular-mass glycoproteins of 230 and 270 kDa are present in *T.* *forsythia*, which are encoded by the *tfsA* and *tfsB* gene, respectively (Higuchi et al. 2000; Lee et al. 2006). The 1,179-amino acid TfsA and the 1,364-amino acid TfsB proteins have a calculated molecular mass of 132 and 154 kDa, respectively, with pI values of 7.8 and 9.9, respectively. Comparison with database entries indicated that the S-layer proteins of *T.* *forsythia* apparently have unique sequences exhibiting no homology to other known S-layer proteins of prokaryotic organisms. Only recently, these S-layer proteins were shown to be covalently modified with identical *O*-glycosidically linked oligosaccharides (Posch et al. 2011).

The exact function of the *T.* *forsythia* S-layer is not yet known, but there are indications that it might be an important virulence factor (Sakakibara et al. 2007; Sharma 2010; Sekot et al. 2011). These include the demonstration that the S-layer mediates adhesion and/or invasion to human gingival epithelial cells (Sakakibara et al. 2007) as well as its potential to delay the recognition of *T. forsythia* by the innate immune system of the host (Sekot et al. 2011). This underlines the importance of the bacterial cell surface in conferring to pathogenicity. For analyzing the role of the *T. forsythia* S-layer proteins in adhesion to and invasion of human gingival epithelial cells, defined insertional inactivation mutants of either of the S-layer genes (named *T. forsythia* Δ*tfsA* and *T.* *forsythia* Δ*tfsB*) as well as an S-layer double mutant (*T.* *forsythia* Δ*tfsAB*) have been constructed (Sakakibara et al. 2007). Results from this study and a more recent study conducted in our laboratory suggest that the S-layer of *T. forsythia* plays an important role in the initiation stage of oral infection, including periodontal disease (Sakakibara et al. 2007; Sekot et al. 2011).

While most S-layer lattices are composed of a single (glyco)protein species (Messner et al. 2010), data from literature indicate that in *T.* *forsythia*, two glycosylated S-layer proteins are present (Higuchi et al. 2000; Lee et al. 2006). In the rare cases of bacterial species which possess more than one S-layer protein, expression of these S-layer proteins is strongly impacted by the growth stage of the bacterium as well as by the physiological and/or environmental conditions. One of the best investigated among those organisms is the Gram-positive bacterium *Bacillus anthracis*, for which regulatory studies have shown that the abundant S-layer proteins EA1 and Sap appear sequentially at the bacterial surface (Couture-Tosi et al. 2002). Within Gram-negative bacteria of the genus *Aquaspirillum*, several species are known to possess two superimposed S-layer proteins. The S-layer ultrastructure is best investigated for *Aquaspirillum serpens* strain MW5 where two, presumably identical, hexagonal S-layer lattices are superimposed (Stewart and Murray 1982). While the underlying layer is attached to the lipopolysaccharide of the outer membrane, the second layer appears to be attached directly to the first layer. Whereas in *A. serpens*, both superimposed S-layers can be clearly distinguished in thin-sectioned bacterial cells, the situation has been less clear for *T.* *forsythia* according to the electron microscopy data published by Sakakibara et al*.* (2007). This poses the interesting questions of how the two different S-layer glycoprotein species of *T.* *forsythia* are arranged, with the principal options for (1) superimposition of two individually assembled S-layers or (2) co-assembly of the two S-layer glycoproteins into a single S-layer. For building up a defined S-layer ultrastructure, it has to be taken into account that the *T.* *forsythia* S-layer proteins are glycosylated (Posch et al. 2011), with glycans naturally occurring in an outward orientation, which would allow them to carry out yet to be identified biological roles.

To assess the potential of the *T.* *forsythia* cell surface in the bacterium–host cross talk the understanding of the S-layer ultrastructure as outermost cell envelope component is essential. Therefore, in this study, different microscopic approaches were applied to characterize the native cell surface of *T.* *forsythia* wild-type and S-layer mutant cells as well as the self-assembly capability of isolated native S-layer glycoproteins (TfsA-GP, TfsB-GP) and their recombinant, glycan-free, counterparts (rTfsA and rTfsB). This included (1) electron microscopic investigations (TEM) of *T.* *forsythia* wild-type and S-layer mutant cells as well as of isolated, purified (glyco)proteins, using either negatively stained, freeze-fractured, freeze-dried, or ultra-thin sectioned samples; (2) immune-fluorescence microscopy of intact bacterial cells using polyclonal antibodies raised against the individual recombinant *T.* *forsythia* S-layer proteins; (3) AFM studies of the S-layer under non-destructive conditions as present on intact bacterial cells as well as visible after recrystallization of recombinant TfsA and TfsB proteins on silica substrates. These microscopic data are interpreted in conjunction with a transcription and translation analysis of the S-layer genes. This study provides the first comprehensive characterization of the cell surface ultrastructure of the oral pathogen *T.* *forsythia*.

## Materials and methods

### Bacterial strains, cultivation, and isolation of native TfsB-glycoprotein

*Tannerella forsythia* ATCC 43037 was purchased from the American Type Culture Collection (ATCC, Manassas, VA, USA). Cultivation of the strains has been described previously (Sekot et al. 2011). Upon insertional inactivation of one or both S-layer genes, the mutant strains *∆tfsA*, *∆tfsB,* and *∆tfsAB* were obtained (Sakakibara et al. 2007). S-layer (glyco)proteins were detected after separation of samples by SDS-PAGE on 8% polyacrylamide (PA) gels upon staining with Coomassie Brilliant Blue R (CBB; GERBU, Wieblingen, Germany) and periodic acid-Schiff (PAS) for protein and carbohydrates, respectively (Posch et al. 2011). A relative quantitation of S-layer proteins was done directly from a CBB-stained SDS-PA gel using an Odyssey scanner (LI-COR, Bad Homburg, Germany) and integration of the intensity signal obtained from the S-layer bands at 700 nm using application software 3.0.

TfsB-GP was harvested from *T.* *forsythia* Δ*tfs*A cells by extraction with HEPES [2-(4-(2-hydroxyethyl)-1piperazinyl)-ethanesulfonic acid] buffer as described previously (Walker et al. 1992; Nomellini et al. 1997; Posch et al. 2011).

### Cloning and expression of TfsA and TfsB, and purification of rTfsA and rTfsB

The *tfsA* and *tfsB* genes were amplified by PCR without their predicted signal sequences using chromosomal *T.* *forsythia* DNA as template. For gene amplification, the primer pairs *tfsA*_23-1179_for/*tfsA*_23-1179_rev and *tfsB*_28-1364_for/*tfsB*_28-1364_rev, respectively, were used (Invitrogen, Lofer, Austria; Table 1). The restriction sites NcoI/NotI were introduced into the for/rev primer for the *tfsA* and the sites BsaI-NcoI/NotI into the for/rev primer for the *tfsB* gene to allow directional cloning of the PCR product into the multicloning site of the expression vector pET28a(+) (Novagen, Darmstadt, Germany) via NcoI/NotI. Since the sequence of the *tfsB* gene contains an NcoI restriction site, a forward primer was designed in such a way that a BsaI site preceded the NcoI site. Thus, upon digestion of the PCR product with BsaI, an NcoI compatible overhang was achieved (Novagen, pET System Manual 11ed; http://lifeserv.bgu.ac.il/wb/zarivach/media/protocols/Novagen%20pET%20system%20manual.pdf). The resulting expression plasmids, pET28a(+)-rtfsA and pET28a(+)-rtfsB, were analyzed by restriction enzyme digestion and finally confirmed by DNA sequencing (AGOWA, Berlin, Germany). The rTfsA and rTfsB proteins are encoded by the sequence from 67 to 3,537 bp (contig TF2661-2662, Oralgen database at: http://www.oralgen.lanl.gov/_index.html) and from 82 to 4,092 bp (contig TF2663), respectively.

**Table 1:** Primers used in this study

Primer	Sequence
*tfsA*_23-1179_for	5′ GGGC↓CATGGATGCGCGACCCTTTTACG 3′
*tfsA*_23-1179_rev	5′ GGGGC↓GGCCGCTTATTTTACTACAGCTTTCACTGCATTC 3′
*tfsB*_28-1364_for	5′ GGGGGTCTCC↓CATGGCACAAATAGCACTGGAGCAAC 3′
*tfsB*_28-1364_rev	5′GGGGC↓GGCCGCTTACTTCACCATCGCTTTTACAGC 3′
Af6	5′ GGGCCATGGATGCGCGACCCTTTTACG 3′
Br6	5′ GGGGCGGCCGCCTTCACCATCGCTTTTACAGC 3′
TfsA_RT2_for	5′ CCTATCACCGATACGGTAAGAG 3′
TfsA_RT2_rev	5′ CCGGATTATCGGAACCTATGG 3′
TfsB_RT1_for	5′ CTGAGCAATGGCGAACAATATCGG 3′
TfsB_RT2_rev	5′ GTTGAGCACTCGGAGTCAAG 3′

Restriction enzyme recognition sites are underlined; restriction sites are indicated by an arrow

*E. coli* BL21-(DE3) (Novagen) was used as host strain for the expression of the two recombinant S-layer proteins. *E. coli* BL21, containing either plasmid pET28a(+)-rtfsA or pET28a(+)-rtfsB, was cultured with continuous shaking at 200 rpm and 37°C for 12 h in Luria–Bertani (LB) medium (Invitrogen) containing 50 mg/L kanamycin. LB medium was inoculated with 1% (v/v) of overnight culture and grown until the optical density at 600 nm (OD_600_) of the culture reached 0.6. Expression of the recombinant proteins was induced by addition of isopropyl-β-d-thiogalactopyranoside (IPTG; Fermentas, St. Leon-Rot, Germany) to a final concentration of 1 mM. Bacteria were harvested 4 h past induction, and proteins were analyzed by SDS-PAGE on 8% PA-gels as described above.

The recombinant S-layer proteins (rTfsA, rTfsB) were isolated from inclusion bodies through ultrasonication (2 min, output 6, 50%, cooled on ice) in lysis buffer (50 mM Tris/HCl, pH = 8.0, 25% saccharose, 1 mM EDTA, 500 units benzonase (Merck, Darmstadt, Germany) and 8 mg lysozyme (Sigma-Aldrich, Vienna, Austria) per g of wet cell pellet). After incubation (15 min at 25°C), samples were centrifuged (6,400 ×*g*, 15 min), and the pellet was washed twice with buffer A (20 mM Tris/HCl, pH = 8.0, 0.2 M NaCl, 1%SDS) and three times with buffer B (10 mM Tris/HCl, pH = 8.0, 0.25% SDS), 20 mL per g pellet, each. Target proteins were extracted in 6 M urea and purified on a Superdex 200, XK20 column (Bio-Rad, Vienna, Austria) using 6 M urea, containing 25 mM ethanolamine, pH = 10.5, as mobile phase.

### qPCR of *T. forsythia* S-layer genes and protein quantification from SDS-PAGE

qPCR for analysis of the *tfsA* and *tfsB* S-layer gene transcription levels in *T.* *forsythia* wild-type cells was performed using isolated RNA (RNeasy Mini Kit; Qiagen, Hilden, Germany) after digestion with DNAseI (Fermentas). RNA was then transcribed into cDNA at the following conditions: 65°C (2 min), 50°C (60 min), and 85°C (5 min). The reaction mixture (40 μL total volume) contained 20 μL PCR-grade water, 4 μL random hexamer primers (Fermentas), 8 μL 5 × reverse transcriptase buffer, 4 μL dNTPs (final concentration 1 mM), 2 μL reverse transcriptase (Fermentas), and 2 μL template RNA isolated from *T.* *forsythia* wild-type cells. qPCR was performed using a Rotorgene 6000 2plex cycler with software version 6.0 (Qiagen). The qPCR mixture of 20 μL was prepared using a SensiMix Plus SYBR qPCR kit (genXpress, Wiener Neudorf, Austria) and consisted of 10 μL 2 × reaction buffer, 7 μL PCR-grade water, 1 μL of each primer (final concentration 0.5 μM), and 1 μL cDNA template. Cycling conditions were as follows: 95°C (10 min), followed by 45 cycles at 95°C (15 s), 55°C (30 s), and 72°C (15 s). Fluorescence was recorded at 470-nm extinction and 510-nm emission. After amplification, a melting curve analysis with a temperature gradient of 0.1°C/s from 65 to 99°C was performed to confirm that only the specific products were amplified. As a standard for quantification, the *tfsAB* operon (amplified from genomic DNA, ~7.6 kbp, primers Af6, Br6; Table 1) was used. Primers applied in *tfsA* qPCR cycling were TfsA_RT2_for and TfsA_RT2_rev; in *tfsB* qPCR cycling, primers TfsB_RT1_for and TfsB_RT2_rev were used (Table 1). Quantification of the transcription levels of *tfsA* and *tfsB* was done applying undiluted and 10-fold diluted cDNA samples. RNA (undiluted) was used to analyze for contaminating DNA after DNAseI treatment to rule out false-positive results.

Relative protein expression levels of TfsA and TfsB in *T.* *forsythia* wild-type cells and in S-layer gene single mutants (*T.* *forsythia* *∆tfsA*, *∆tfsB*) were analyzed after CBB staining as described above. Gels were loaded with standardized amounts of crude cell extracts prepared from the respective *T.* *forsythia* cells.

### Raising, purification, and labeling of polyclonal antibodies against *T.* *forsythia* S-layer proteins

Purified rTfsA and rTfsB proteins, respectively, were dialyzed against 18 MOhm-water (MilliQ water; Millipore, Vienna, Austria), lyophilized and resuspended in PBS (phosphate-buffered saline) containing 0.1% SDS at a concentration of 0.85 mg/mL and used to raise polyclonal antibodies in rabbits. Antisera were tested by Western blot analysis for cross-reactivity prior to use. Pure antibody fractions were received upon HiTrap^™^Protein G 2 mL HP (GE Healthcare, Uppsala, Sweden) chromatography after washing with 20 mM sodium phosphate buffer (pH = 7.0). Elution was done with 0.1 M glycine solution (adjusted to pH = 2.7 with HCl), collecting 1-mL fractions into tubes containing 40 μL of 1 M Tris solution. Antibodies were concentrated by centrifugation with Amicon^®^ Ultra 3 K centrifugal filters (Millipore) and stored in 20 mM sodium phosphate buffer (pH = 7.0). Fab fractions were produced by papain digestion using the Pierce^®^ Fab Micro Preparation Kit (Thermo Scientific, Waltham, MA, USA) and labeled with Pierce^®^ FITC (fluorescein isothiocyanate) Antibody Labeling Kit (Thermo Scientific) according to the manufacturer’s instructions.

### Ultra-thin sectioning, negative-staining, freeze-etching, and freeze-drying experiments and transmission electron microscopy

Ultrathin-sectioning of *T. forsythia* cells was carried out as described previously (Messner et al. 1984). Briefly, the room-temperature processing included fixation of samples with 2.5% (w/v) paraformaldehyde/2.5% (w/v) glutaraldehyde/0.5% (w/v) tannin in cacodylate buffer and osmium tetroxide fixation without ruthenium red. Dehydration was performed in an increasing alcohol series before samples were embedded in Epon resin. Negative staining of crystallized S-layer proteins as well as of intact *Tannerella* cells was performed with 1% uranyl acetate solution for 30 s (Messner et al. 1986).

Freeze-etching of intact bacteria was carried out in different freeze etching units, including a BAF 400-T unit (BAL-TEC, Balzers, Liechtenstein), a BAF 060 unit (Leica, Wetzlar, Germany), and a Cressington CFE-50 unit (Cressington, Watford, UK). Fracturing and etching of frozen samples was done at −95°C for 90 s prior to Pt/C shadowing. Replicas were purified for 30 min in 70% sulfuric acid, then neutralized in water and subsequently subjected to 14% sodium hypochloride solution (for 3–5 min), followed by three washing steps in distilled water and immobilization on 400-mesh TEM copper grids (Agar Scientific, Stansted, UK).

For freeze-drying, bacteria were resuspended in 50 mM Tris/HCl buffer (pH = 7.2), disrupted with a Branson model 250 sonifier (Branson Ultrasonics, Danbury, CT, USA) for 40 s with 50% output (no cooling), and centrifuged (1,300×*g*, 10 min). The pellet was resuspended in 50 mM Tris/HCl buffer (pH = 7.2, containing 1% Triton X-100), incubated for 20 min at 25°C, and centrifuged. After two further washing steps with Tris/HCl buffer (pH = 7.2), the pellet was resuspended in water. For adsorption of disrupted bacterial cells, a freshly prepared Formvar and carbon-coated 300-mesh TEM copper grid (Agar Scientific) was floated facedown for 1 min on one drop of bacterial suspension. After removal of excess of liquid from the grid with filter paper, it was rapidly frozen in nitrogen-cooled liquid CHClF_2_ and immediately loaded onto a nitrogen-cooled specimen holder for freeze-drying in the BAF 060 unit (Leica). Freeze drying was performed at −80°C for 2 h as described previously (Messner et al. 1986).

All samples were investigated in a Tecnai G^2^ 20 Twin transmission electron microscope (TEM; FEI, Eindhoven, The Netherlands), operating at 80 keV. Pictures were taken with an FEI Eagle 4 k CCD camera (4,096 × 4,096 pixels). The magnification was calibrated by using negatively stained catalase crystals (Wrigley 1986). Image processing was done with software developed in house based on Fourier domain techniques according to Amos et al. (1982) and Crowther et al. (1996). S-layer lattice parameters were obtained from the power spectra.

### Immunofluorescence staining and fluorescence microscopy of *T.* *forsythia* wild-type cells

*T. forsythia* wild-type and mutant cells were washed twice with PBS buffer and unspecific-binding sites were blocked (1 h, 25°C, constant stirring) in PBS containing 5% BSA (blocking solution). Bacteria were exposed to primary polyclonal rabbit anti-TfsA antiserum diluted 1:20 in blocking solution (1 h, 25°C, constant stirring). After removal of unspecific binders by washing the bacteria three times with Tris-buffered saline containing 1% Tween-20 (TBST), the secondary anti-rabbit tetramethyl rhodamine isothiocyanate (TRITC)-conjugate antibody (Sigma-Aldrich) was diluted to a final concentration of 0.15–0.3 mg/mL with blocking solution and added to the bacteria, followed by exposure for 1 h at 25°C under constant stirring. After three washing steps with TBST and one step with PBS, bacteria were resuspended in PBS and analyzed with an Eclipse TE2000-S fluorescence microscope (Nikon, Vienna, Austria). Pictures were taken with a DS-Qi1 Mc-camera (Nikon), and the same image section was recorded in bright-field phase contrast, as well as in the TRITC channel, the respective overlay was generated with NIS Elements BR software (Nikon).

### Atomic force microscopy (AFM) of *T.forsythia* wild-type cells

*T. forsythia* wild-type cells were washed twice in PBS and immobilized by mechanical trapping on 0.8-μm polycarbonate membranes (Millipore) for non-invasive in vivo imaging (Dupres et al. 2009). After filtration of a washed bacterial suspension, the filters were gently rinsed with PBS buffer and attached to the sample holder using a double-side adhesive tape, and the mounted sample was measured in the AFM liquid cell. All AFM measurements were performed in PBS buffer and in deionized water, using a commercial Agilent 5500 AFM (Agilent Technologies, Chandler, AZ, USA). Magnetically coated AFM cantilevers with a nominal spring constant of 0.1 N/m were used for magnetic AC mode (MAC mode) imaging (Tang et al. 2008). The resonance frequency of the cantilever was selected between 9 and 11 kHz in liquid, and the measurement frequency was set to 20% below the resonance frequency. The Gwyddion image viewer open source program was used to analyze topographic images of the surface, as well as polynomial flattening analysis.

### Self-assembly experiments

Recombinant S-layer protein solutions (rTfsA, rTfsB) as well as native TfsB-GP were dialyzed against different buffers, including 0.5 mM Tris/HCl (containing 10 mM CaCl_2_, 10 mM MgCl_2_), PBS, HEPES buffer, citric acid, to cover the pH values of 3.0, 4.0, 5.0, 6.5, 7.0, 7.5, 8.0, 8.5, and 9.0, as well as against MQ and tap water. Samples were subsequently applied to Formvar and carbon-coated 300-mesh TEM grids (Agar Scientific) and negatively stained with 1% uranyl acetate (see “Ultrathin sectioning, negative-staining, freeze-etching, and freeze-drying experiments and transmission electron microscopy”).

For AFM experiments, the rTfsA and rTfsB proteins alone, an equimolar mixture of those as well as native TfsB-GP were dialyzed against MQ water; the protein monomers were diluted in crystallization buffer (10 mM CaCl_2_, 0.5 mM Tris/HCl, pH = 8.0) to a final concentration of 1–10 mg/mL and applied to silicon wafers. For the rTfsB protein and TfsB-GP, the silicon wafer was additionally coated for 20 min with 1 mg/mL polyethylenimine (PEI) in distilled water. Crystallization was carried out for 12 h at 25°C, and AFM measurements were performed in contact mode with a Nanoscope IIIa multimode (Veeco Instruments, Santa Barbara, CA, USA) with a J-scanner (nominal scan size 130 mm). Scanning of samples was performed at approximately 4 Hz in 100 mM NaCl to avoid electrostatic repulsion between tip and sample, and the applied force during scanning was minimized to prevent the tip from modifying the sample. For optimal resolution, standard oxide-sharpened silicon nitrite cantilevers with a nominal spring constant of 0.06 N/m (NP-S, NanoProbes, Digital Instruments, Santa Barbara, CA, USA) were used for imaging. The Nanoscope 5.0 imaging software (Veeco) was used to analyze and process (first order flattening) topographic images of the surface.

## Results

### Detection of *T. forsythia* S-layer (glyco)proteins in cell extracts

**Fig. 1:**
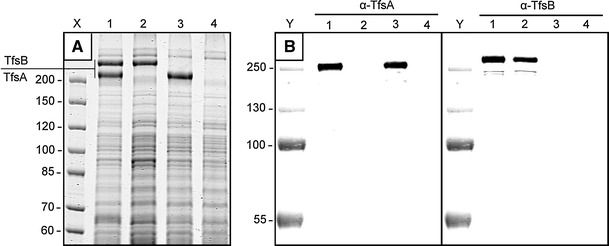
**a** CBB-stained SDS-PAGE of crude cell extracts from *T. forsythia* wt and S-layer mutants used in this study. Lane X, PageRuler Unstained Protein Ladder (Fermentas); *lane* 1, *T. forsythia* wt; *lane* 2, *T.* *forsythia* Δ*tfsA*; *lane* 3, *T. forsythia* Δ*tfsB*; *lane* 4, *T. forsythia* Δ*tfsAB*. **b** Western blot of whole cell extracts from *T.* *forsythia* wt and S-layer mutants (same order as in **a**) probed with antibodies against rTfsA and rTfsB, respectively. Lane Y, Page Ruler Prestained Protein Ladder (Fermentas)

The presence of the two S-layer glycoproteins TfsA-GP and TfsB-GP was demonstrated by SDS-PAGE of SDS-solubilized whole-cell extracts upon CBB protein staining (Fig. 1a) and PAS staining for carbohydrates (Posch et al. 2011), with these S-layer glycoproteins corresponding together to approximately 10% of whole cellular protein according to intensity signal integration. Western blotting using the polyclonal rabbit antibodies raised against the individual S-layer proteins corroborated the presence of the two glycoproteins TfsA-GP and TfsB-GP of *T.* *forsythia* exhibiting apparent molecular masses of 230 and 270 kDa, respectively, with the antibodies showing no cross-reactivity (Fig. 1b). In the deletion mutants *T.* *forsythia* Δ*tfsA*, Δ*tfsB*, and Δ*tfsAB*, the respective bands were missing (Fig. 1b).

### Transcription of *tfsA* and *tfsB* genes and S-layer protein quantification

**Fig. 2:**
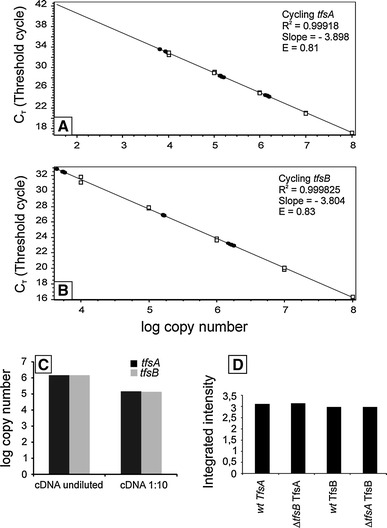
Standard curves for the quantification of **a** *tfsA* and **b** *tfsB* gene transcription in *T.* *forsythia* wt cells. Standard values (*open boxes*) were obtained by amplifying the *tfsA* or *tfsB* genes using different dilutions of the *tfsAB* operon (purified and quantified PCR product used as template) in ascending order. *Black circles* indicate calculated C_T_ values for analyzed samples. **c** Absolute quantification of *tfsA/tfsB* gene transcription in *T.* *forsythia* wt cells. Undiluted and 10-fold diluted cDNA was used as template and consistent absolute transcription levels (copy numbers) were determined for both genes. **d** Relative protein expression levels of TfsA/TfsB in *T.* *forsythia* wild-type cells and respective mutant strains. 0.7 mg of total crude cell extract applied to each lane in SDS-PAGE

qPCR was applied to determine the transcription levels of the S-layer genes *tfsA* and *tfsB* in *T. forsythia*. Absolute quantification was performed by calculating standard curves for both genes (Fig. 2a, b). The absolute copy numbers of *tfsA* and *tfsB* from a cDNA preparation were determined from the corresponding standard curves using the C_T_ (cycle threshold) values. As the results suggest, both *tfsA* and *tfsB* genes, which form a putative operon structure on the *T.* *forsythia* genome (Lee et al. 2006), are uniformly transcribed into mRNA (Fig. 2c). For both genes, undiluted and 10-fold diluted samples gave comparable absolute copy numbers. Minor contamination of the cDNA template with genomic DNA was detected, but the overall influence on the experiment was negligible (data not shown). Melting curve analysis of both amplified products confirmed the specificity of the respective PCRs (data not shown).

Semi-quantification of the protein expression levels of TfsA-GP and TfsB-GP from *T. forsythia* wild-type cells and S-layer mutants after separation on an SDS-PA gel (compare with Fig. 1a) corroborated the qPCR results (Fig. 2d). Using *T. forsythia* wild-type cells, both S-layer proteins exhibited a comparable intensity signal. Interestingly, the intensity signals obtained for TfsA-GP and TfsB-GP glycoproteins from S-layer single mutants were comparable to those from wild-type cells, where both S-layer glycoproteins are present. Thus, deletion of one of the S-layer genes does not influence the overall expression level of the respective other protein, ruling out the option that one S-layer protein might replace the other in the mutant for formation of the mature S-layer lattice.

According to these data, the S-layer genes *tfsA* and *tfsB* are both transcribed into mRNA and expressed into protein in equal ratios. However, based on this analysis, it cannot be discerned whether a single S-layer would form upon co-assembly of the two S-layer glycoproteins species (TfsA-GP, TfsB-GP) or whether the two species would assemble into two independent, superimposed monolayers.

### Visualization of the native S-layer lattice on thin-sectioned, negatively stained, freeze-etched, and freeze-dried preparations of *T. forsythia* cells

**Fig. 3:**
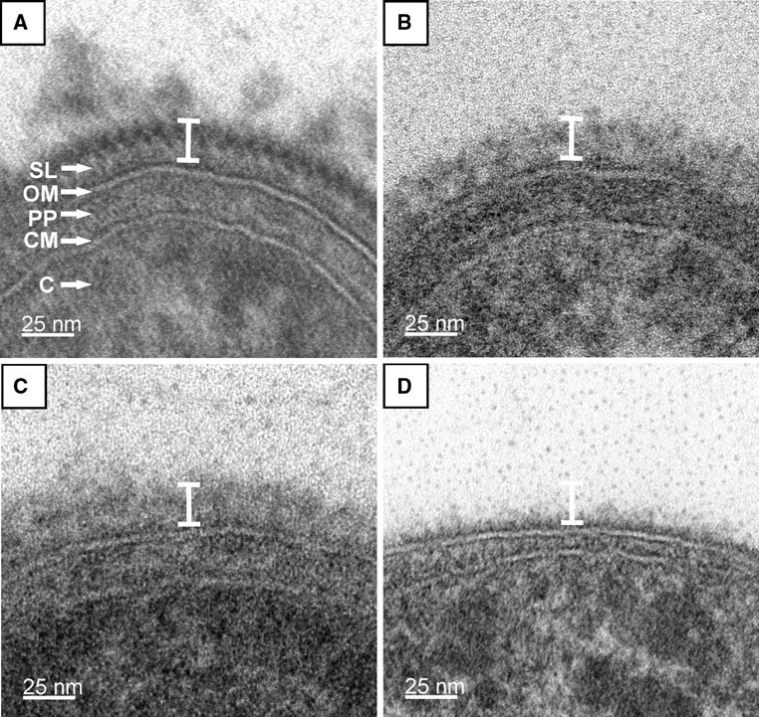
Ultra-thin cross-sections of whole cell preparations from *T.* *forsythia*. **a** *T. forsythia* wt cells with a 2D crystalline S-layer; **b** *T.* *forsythia* Δ*tfsB* mutant cell; **c** *T. forsythia* Δ*tfsA* mutant cell. In either S-layer single mutant the S-layer is not visible as a cross-sectioned 2D-crystal, but an unstructured “stained mass” without periodicity is present on top of the outer membrane, which is less densely packed than the S-layer on wild-type cells. **d** *T. forsythia* Δ*tfsAB* double mutant without an S-layer. The *white bar* indicates that the overall thickness of the S-layer is identical in *T.* *forsythia* wt and *T.* *forsythia* S-layer single-mutant cells. *SL* S-layer, *OM* outer membrane, *PP* periplasmic space, *CM* cytoplasmic membrane, *C* cytoplasm

TEM analysis of the S-layer on *T.* *forsythia* wild-type cells as well as on the S-layer deletion mutant cells was first performed by ultra-thin sectioning (Fig. 3a–d).

Typically, *T.* *forsythia* wild-type cells are approximately 3.5 ± 1 μm in length and 0.6 ± 0.1 μm in diameter (not shown). They possess a typical Gram-negative cell envelope profile consisting of a cytoplasmic membrane, a periplasm, and an outer membrane. Additionally, this outer membrane is completely covered by an S-layer with a thickness of approximately 22 nm, as determined from thin sections (Fig. 3a). Our data from cross-sectioned *T.* *forsythia* wild-type cells show a continuous, cross-sectioned S-layer, defined as a 2D protein crystal, in a strikingly clear way (Fig. 3a). The cells in which one of the S-layer proteins was deleted, *T.* *forsythia* Δ*tfs*A and *T.* *forsythia* Δ*tfs*B, respectively, the S-layer as such is not visible (Fig. 3a, b). Instead, an additional “gray mass” without any periodicity (compare with the zigzag in Fig. 3a), which would reflect the presence of a 2D crystalline arrangement of the S-layer protein, is present on top of the outer membrane with a similar width as the S-layer on the wild-type cells. The *T.* *forsythia* Δ*tfs*AB cells (Fig. 3d) are the devoid of any “stained mass” outside of the outer membrane (compare with Fig. 1a, lane 4).

**Fig. 4:**
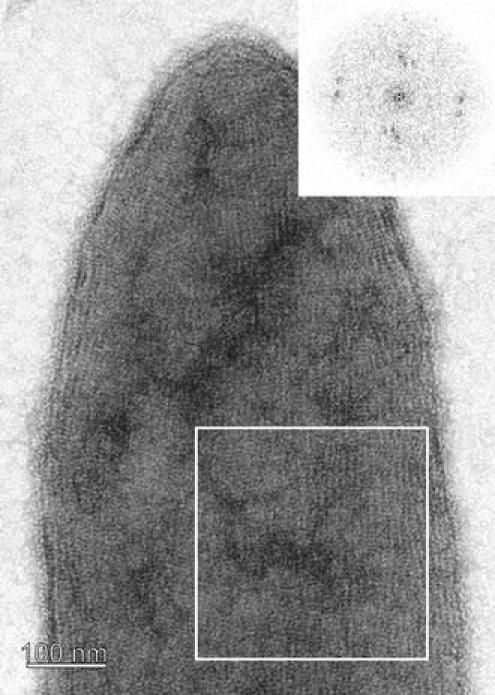
Negative-staining of a *T.* *forsythia* whole cell preparation*.* During adsorption of the cell to the EM grid parts of the cell were flattened so that the cytoplasm was squeezed away and, therefore, the “*upper*” and “*lower*” S-layer came into close distance. The *inset* shows the resulting power spectrum

A first impression of the regularity of the *T.* *forsythia* S-layer lattice in a 2D projection was obtained after negative-staining of *T.* *forsythia* wild-type cells. A representative image exhibiting the regular array of glycoproteins was obtained from those areas of the cell where, due to the adsorptive attachment to the grid, an almost complete flattening of the cell body took place leaving the “top” and the “bottom” S-layer in close vicinity (Fig. 4). The power spectrum of the resulting *Moiré* pattern (Glauert 1966; Valamanesh et al. 2011) confirmed that in these preparations, two single S-layers are lying on top of each other (Fig. 4, inset). This analysis already indicates that a superimposition of two single S-layers on intact cells is unlikely, since this would have resulted in four superimposed monolayers in this negatively stained preparation, given that the uranyl acetate staining solution can penetrate between the double layers. Fourier domain image analysis was used to determine both the lattice symmetry and the lattice constant of the S-layer lattice on the wild-type organism. For the negatively stained samples, an S-layer lattice with square symmetry exhibiting an average (*n* = 3) lattice constant of 10.3 ± 0.7 nm, with γ = 90° ± 4.3° was determined. However, neither of the negatively stained preparations of the *T.* *forsythia* S-layer single-mutant cells showed a regular S-layer lattice in the TEM analysis, which lead us to speculate about a scenario of co-assembly of TfsA-GP and TfsB-GP to form the native S-layer lattice as present on the *T.* *forsythia* wild-type organism.

**Fig. 5:**
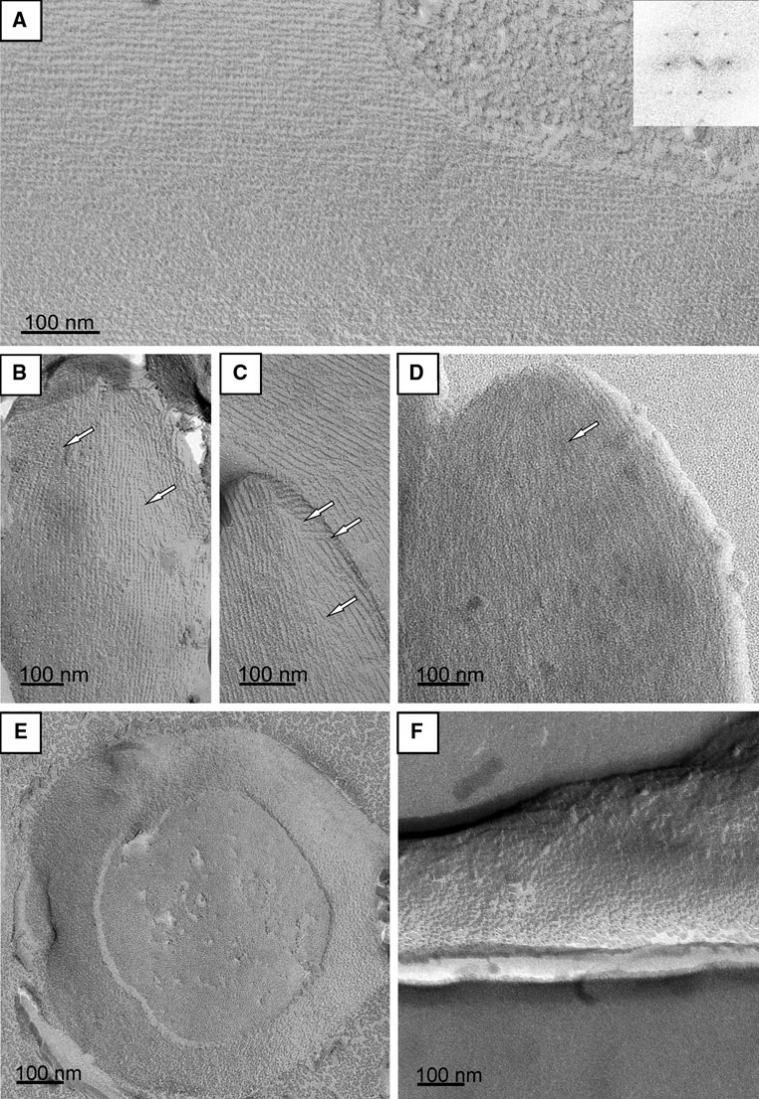
Freeze-etched (**a–c**, **e**, **f)** and freeze-dried (**d**) metal-shadowed preparations of *T.* *forsythia* wt cells. **a** Cylindrical portion of the cell showing an undisturbed square S-layer lattice; *inset*, power spectrum of this lattice. **b**, **c** Cell the poles with grain boundaries in the S-layer lattice (*white arrows*, compare also with **d**). **e** Cell pole of a freeze-dried *T. forsythia* wt cell. S-layer lattice on (**e**) *T.* *forsythia* Δ*tfsA* and **f** *T. forsythia* Δ*tfsB*

Analysis of the ultrastructure of the cell surface of *T.* *forsythia* was also carried out by freeze-etching (Fig. 5a, b) and freeze-drying experiments (Fig. 5c). In both preparations, the wild-type cells exhibited a similar surface pattern for the cylindrical part of the cell envelope (Fig. 5a) as determined by negative-staining (Fig. 4), reflecting the architecture of a square S-layer lattice (Fig. 5a). Fourier domain image analysis confirmed the presence of a square lattice on the cell surface of *T.* *forsythia* wild-type cells with an average (*n* = 5) lattice constant of 10.3 ± 0.3 nm and γ = 90° ± 5.0° according to the freeze-etching experiments. A rare view onto the region of the cell pole of a single cell showed grain boundaries between different S-layer crystallites (Fig. 5b) as they have already often been observed on other S-layer-covered cells (Sleytr 1978; Pum et al. 1991; Kingl et al. 2011). In freeze-dried preparations, only *T.* *forsythia* wild-type cells showed a clearly recognizable S-layer lattice with average (*n* = 5) lattice parameters of 10.8 ± 0.5 nm and γ ~ 90° ± 3.0°, which is in agreement with the data from freeze-etching and negative-staining experiments (see above).

While visualization of the S-layer lattice was straightforward on the cell surface of the wild-type organism upon freeze-etching, this could be achieved only on a few cells of the S-layer single mutants *T.* *forsythia* Δ*tfsA* and Δ*tfsB*; with the lattice being less clearly visible; this confirmed the results from the negative staining experiments. These mutant cells showed a faint regular surface structure with lattice parameters of 8.6 nm x 8.7 nm and γ ~ 90° for *T.* *forsythia* Δ*tfsA* cells (Fig. 5e) and 9.7 nm × 8.7 nm and γ ~ 90° for *T.* *forsythia* Δ*tfsB* cells (Fig. 5f). Thus, obviously none of the mutant cell S-layer proteins alone is capable of forming a lattice identical to that on native cells, although both still possess some residual self-assembly capability.

**Fig. 6:**
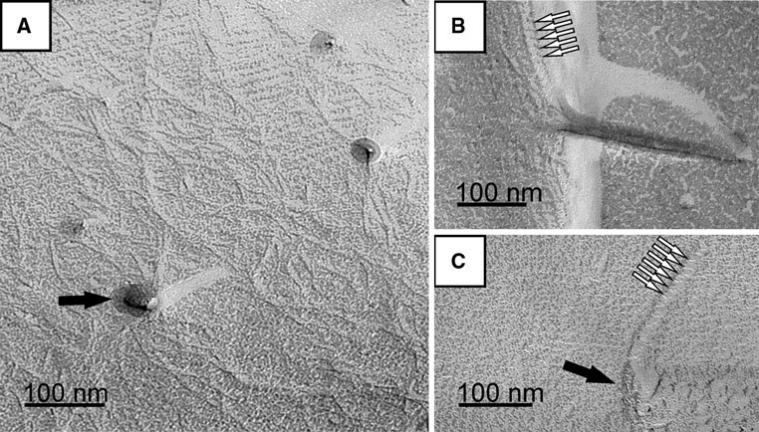
Evidence of surface appendages on *T.* *forsythia* wt cells upon freeze-etching. **a** Filamentous pilus-like structures, at certain regions of the *T.* *forsythia* cell surface that, have been reattached onto the S-layer lattice during the sublimation of ice in the freeze-etching process. **b** Fragment of a sheared flagellum, reattached to the S-layer and **c** sheared flagellum with hook region. *Black arrows* indicate the insertion site of a hook region of a sheared flagellum (**a**, **c**), *white arrows* indicate the pitch of the flagellins (**b**, **c**)

Although *T.* *forsythia* has been described as a non-motile bacterium (Tanner et al. 1986; Sharma 2010), we found occasionally flagella with diameters of approximately 20 nm and pilus-like structures, obviously randomly distributed over the S-layer surface during the sublimation of ice in the freeze-etching experiments (Fig. 6a–c). In addition, there is evidence of the presence of hook regions as required for anchoring the flagella to the outer membrane (Fig. 6a, c). The number of the presumably peritrichous flagella on *T.* *forsythia* cells seems to be low, whereas the pilus-like structures occur more frequently.

### AFM imaging of bacterial cells

**Fig. 7:**
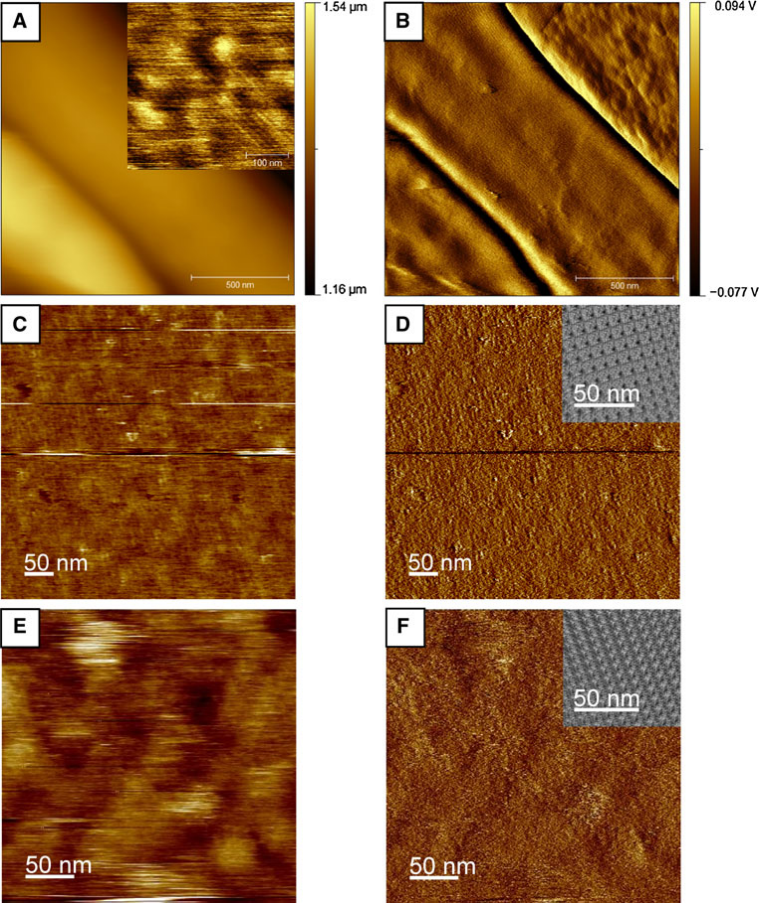
AFM **a** topography and **b** amplitude image of *T. forsythia* wild-type cells. The *inset* in **a** shows a zoomed part of the bacterial surface. AFM analysis of in vitro self-assembly experiments of the recombinant S-layer proteins rTfsA (**c**, **d**) on silicon and of rTfsB (**e**, **f**) on PEI-coated silicon. **c**, **e** Height images and **d**, **f** deflection images and image reconstruction of the 2D crystalline S-layer lattice (*insets*) of rTfsA (**d**) and of rTfsB (**f**)

**Fig. 8:**
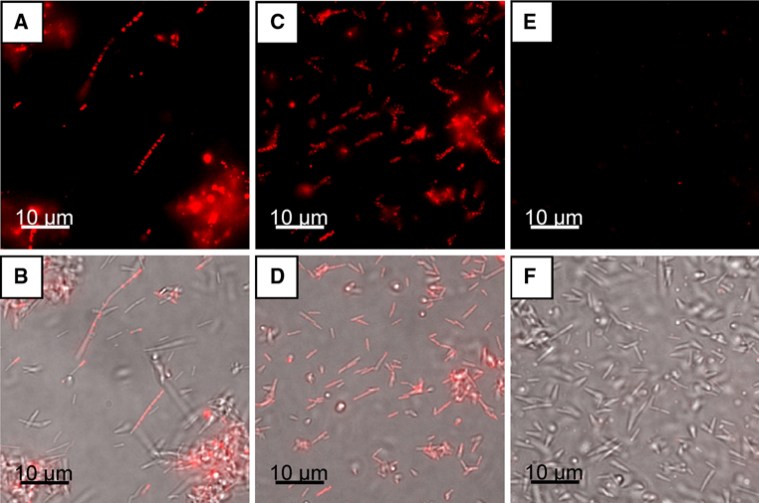
Immunofluorescence images of *T.* *forsythia* wild-type (**a**, **b**), *T.* *forsythia* ∆*tfsB* (**c**, **d**) and *T.* *forsythia* ∆*tfsA* (**e**, **f**), displaying the outermost localization of TfsA on the bacterial cell surface upon probing with TRITC-conjugated anti-rTfsA antibody. The *upper row* (**a**, **c**, **e**) displays the TRITC channel and the *bottom row* (**b**, **d**, **f**) shows the respective overlaid pictures of the bright field phase contrast light micrograph and the TRITC channel

Scanning probe microscopy was used in the MAC mode to image the cell surface of *T. forsythia* wild-type cells by investigating the S-layer ultrastructure. Cells were trapped within a filter (Dufrêne 2002), because this method appears promising as a generally applicable immobilization method for bacteria of various shapes and sizes. Since it does not require exposure to chemicals, or drying, it does not affect cell viability or trigger undesirable biological responses. AFM height and amplitude images of *T. forsythia* wild-type cells are shown in Figure 7. Because of the large curvature of the bacterial cells, the topographic image (Fig. 7a) has fairly poor resolution, whereas the amplitude image (Fig. 7b) is much more sensitive to changes in the surface topography. The inset in Figure 8a was flattened with a twelfth-order polynomial. The latter image shows that native *T. forsythia* wild-type cells are featured with a periodic square lattice structure of a center-to-center spacing in the range of 9.1 ± 0.8 nm and an angle of γ ~ 90.0° ± 4.8°. These values are in agreement with the TEM analyses described above.

### Immunofluorescence staining of S-layer proteins on *T.* *forsythia* cells

To prove our hypothesis of the presence of both S-layer glycoproteins on the cell surface of *T.* *forsythia* wild-type cells, immunofluorescence microscopy in conjunction with the polyclonal antibodies raised against the individual rTfsA and rTfsB proteins was used. Prior to this experiment, the purified antibodies were shown to recognize and bind the respective target protein and to possess no cross-reactivity in Western blot analysis (Fig. 1b).

The presence of the TfsA-GP could be imaged by a fluorescence signal derived upon probing *T. forsythia* wild-type and *T. forsythia* ∆*tfsB* cells with a TRITC-conjugated anti-rTfsA antibody (Fig. 8a–d), while no signal was obtained from the *T. forsythia* ∆*tfsA* and *T.* *forsythia* ∆*tfsAB* control cells (Fig. 8e, f). Surprisingly, the presence of the TfsB-GP could not be visualized in an identical experimental setup using the corresponding TRITC-conjugated anti-rTfsB antibody (not shown). To exclude the possibility that due to steric hindrance the antibody could not access the TfsB protein, which we assumed to be part of the co-assembled S-layer lattice, the experiment was repeated using FITC-conjugated Fab fragments of the TfsB antibody. However, again, no signal was obtained with either *T.* *forsythia* wild-type or *T. forsythia* ∆*tfsA* cells, and also the controls were negative.

These data allow different interpretations: (1) since the denaturated rTfsB protein was used for raising antibodies, it might well be that the antibody does not recognize the native epitopes of the folded S-layer protein as present on the cell surface; and (2) the recognition sites of the folded S-layer protein are not accessible for the antibody, because they might be orientated toward the underlying outer membrane or buried within the S-layer.

### Re-assembly experiments of native and recombinant *T. forsythia* S-layer (glyco)proteins

Although the microscopic investigations of native *T.* *forsythia* cells already indicated that most likely co-assembly of the TfsA-GP and the TfsB-GP does occur under native conditions, re-assembly experiments were also carried out in vitro, using purified S-layer glycoproteins as well as the recombinant S-layer proteins rTfsA and rTfsB, since both the S-layer proteins as well as the glycosylated forms, per definition, should possess inherent self-assembly capability.

For most of the chosen conditions (varying buffers, molarities, and pH values), self-assembly of individual native and recombinant *T. forsythia* (glyco)proteins could not be demonstrated in vitro according to analyses of negatively stained preparations. Only upon dialysis either against Tris/CaCl_2_ buffer (pH = 8.0) or tap water, the (glyco)proteins were prone to impaired self-assembly with exclusively multilayered stacks of (glyco)proteins being formed, with *Moiré* patterns displaying striations of different width and different angles to each other, which cannot be clearly traced back a specific lattice symmetry (not shown). A comparable result was obtained when an equimolar mixture of native or recombinant *T. forsythia* (glyco)proteins, respectively, was analyzed for self-assembly behavior, attempting to mimic the most likely native situation (data not shown). Although it is generally known that covalently bound glycans, as present on the native *T.* *forsythia* proteins, can influence proper protein folding and, consequently, self-assembly, our results suggest that self-assembly of *T. forsythia* S-layer (glyco)proteins is not affected by the glycosylation status.

The impaired self-assembly capability of the S-layer (glyco)proteins in vitro was also observed in the AFM studies. For these approaches, a monomer solution obtained by dialysis of a solution of the recombinant S-layer proteins rTfsA and rTfsB as well as of TfsB-GP against distilled water was used (Chung et al. 2010). In the AFM measurements, most of the recombinant S-layer protein was found to adsorb in the form of unstructured patches on the chosen support. Again, as a rare event, for rTfsA, an S-layer lattice with 13.9 nm × 12.0 nm and γ = 80° could be resolved on a silica support (Fig. 7c, d), while for rTfsB, a regular protein lattice with the parameters of 10.0 nm × 10.0 nm and γ = 73° could be detected on a PEI-coated silicon surface (Fig. 7e, f). The same result was obtained for the corresponding glycoprotein TfsB-GP (not shown). It is important to note that the PEI coating of the support did not reveal any regular features (not shown). When co-assembly of rTfsA and rTfsB was attempted, exclusively unstructured patches were seen on the AFM support (not shown).

Generally, the AFM findings support the data from the negative staining and low-temperature preparation method experiments, indicating a strongly impaired self-assembly capability of *T.* *forsythia* S-layer proteins in vitro under the chosen conditions and/or a certain degree of structural flexibility of the individual native and recombinant *T.* *forsythia* S-layer (glyco)proteins alone after self-assembly.

## Discussion

Periodontitis is initiated due to colonization of the oral cavity by a group of Gram-negative anaerobes in the form of a subgingival biofilm. The disease progresses as a result of the direct effects of bacterial virulence factors on host tissues, as well as self-damaging host responses to the colonizing bacteria (Socransky et al. 1998). Among the few putative virulence factors that have been identified in the oral pathogen *T. forsythia* are two components which, due to their localization on the bacterial cell surface, can be expected to be involved in initial stages of the bacterium–host cross talk; these are a leucine-rich repeat cell surface-associated and cell surface-secreted protein BspA (Sharma et al. 1998) and the S-layer (Sabet et al. 2003; Sakakibara et al. 2007; Sekot et al. 2011). This study focused on the ultrastructural characterization of the S-layer of *T.* *forsythia* as outermost cell surface layer by a set of microscopic, biochemical, and genetic methods, aiming at the determination of S-layer lattice parameters. *T.* *forsythia* presents a situation so far unknown for Gram-negative bacteria, which is the presence of two S-layer proteins that are additionally posttranslationally modified with so far unique *O*-glycosidically linked oligosaccharides (Posch et al. 2011).

In the course of this study, it was demonstrated that the S-layer genes *tfsA* and *tfsB* are both transcribed into mRNA and expressed into protein in equal amounts, leaving the option for superimposition of two S-layers or co-assembly of TfsA-GP and TfsB-GP into a single S-layer lattice that covers the *T.* *forsythia* cells. While TEM analysis of wild-type cells revealed the presence of a square S-layer lattice with average lattice parameters of 10.3 ± 0.3 nm and γ = 90° ± 5.0° (freeze-etching) and of 10.3 ± 0.7 nm and γ = 90° ± 4.3° (negative staining), a more blurred lattice structure with a smaller lattice constant of approximately 9.0 nm (freeze-etching) was found on some surface areas of those *Tannerella* cells that were deficient in expressing either of the two S-layer glycoproteins (*T. forsythia* ∆*tfsA*, *T. forsythia* ∆*tfsB*). We interpret this slight decrease in the lattice constant as a consequence of the individual S-layer glycoproteins assuming a different conformation depending on the composition of the S-layer, that is, “mono-species” S-layer versus “co-assembled” S-layer lattice.

Despite the fact that we do not have a final immunological proof for the presence of the TfsB-GP on the cell surface of *T.* *forsythia* wild-type cells, based on the results from the TEM investigations, we favor the assumption of TfsA-GP and TfsB-GP forming the mature S-layer lattice by equimolar co-assembly. This is vaguely supported by previous studies by others, where the presence and accessibility of the TfsB-GP on *T.* *forsythia* wild-type cells was shown by TEM using a gold-conjugated anti-TfsB-GP antibody (Higuchi et al. 2000; Moriguchi et al. 2008; Sakakibara et al. 2007). However, since that antibody also reacted with the native TfsA-GP, it might be that it preferentially recognized the S-layer glycans on the proteins, which we could show to be identical (Posch et al. 2011).

While the impaired self-assembly capability of the recombinant S-layer proteins might be explained by the fact that the chaotropic agent (5 M guanidine hydrochloride) used for their isolation causes irreversible conformational changes that might prevent, subsequently, proper self-assembly, no comparable stress is exerted on the S-layer glycoproteins of the intact *T.* *forsythia* S-layer single mutants. This together with the results from in vitro self-assembly studies indicate an impaired self-assembly capability of the individual S-layer (glyco) proteins, which makes them distinctly different from most other known S-layer (glyco)proteins (Toca-Herrera et al. 2005; Messner et al. 2010), which usually retain their self-assembly capability in vitro.

Another finding of this study concerns, for the first time, the visualization of flagella and pilus-like structures on *T. forsythia* cells. Frequently, bacteria and archaea expose on their outer surfaces a variety of thread-like proteinaceous organelles with which they interact with their environments (Ghosh and Albers 2011; Sauer et al. 2000). These structures are repetitive assemblies of protein subunits and serve different roles in cell motility, adhesion and host invasion, protein and DNA secretion and uptake, conductance, or cellular encapsulation. With regard to oral bacteria, adherence to a surface is a key element for colonization of the human oral cavity (Whittaker et al. 1996). Thus, it is conceivable that these structures contribute to the pathogenicity potential of *T.* *forsythia*. There are many pathogens known for which, especially glycosylated, flagella mediate virulence-associated phenomena (Thibault et al. 2001; Schirm et al. 2003). Considering the so far unique glycosylation of the S-layer proteins TfsA and TfsB from *T.* *forsythia* (Posch et al. 2011), one might speculate about glycosylation of these surface appendages. Such a situation is, for instance, known from archaea, where S-layer proteins and flagella are decorated with the same glycan structures (Voisin et al. 2005; Jarrell et al. 2010, 2011). Consequently, these surface appendages might be of high importance for future studies with *T. forsythia*.

**Fig. 9:**
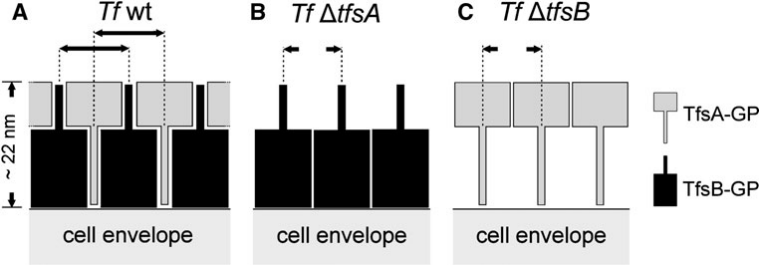
Scheme of the possible arrangement of the S-layer glycoprotein species within the square S-layer lattice of *T.* *forsythia* wild-type (**a**) and S-layer single-mutant cells (**b**, **c**). Scheme drawn not to scale

Summarizing, the most likely situation on *T. forsythia* wild-type cells is the presence of the two intercalating S-layer glycoproteins TfsA-GP and TfsB-GP, forming a square S-layer lattice (Fig. 9a). The slight reduction in the lattice constant of the S-layer lattices present on the individual S-layer gene mutants might be explained by a denser packing of the protomers when the space-filling counterpart is missing (Fig. 9b, c). It is conceivable that this so far unique ultrastructure of an S-layer is of relevance for the complex reactions occurring during the bacterium–host cross talk, such as the adhesion in the oral cavity under physiological as well as pathological conditions.
